# Spontaneous BOLD Signal Fluctuations in Young Healthy Subjects and Elderly Patients with Chronic Kidney Disease

**DOI:** 10.1371/journal.pone.0092539

**Published:** 2014-03-20

**Authors:** Hesamoddin Jahanian, Wendy W. Ni, Thomas Christen, Michael E. Moseley, Manjula Kurella Tamura, Greg Zaharchuk

**Affiliations:** 1 Department of Radiology, Stanford University, Stanford, California, United States of America; 2 Department of Electrical Engineering, Stanford University, Stanford, California, Untied States of America; 3 Geriatric Research and Education Clinical Center, Palo Alto Veterans Affairs Health Care System and Division of Nephrology, Stanford University, Stanford, California, United States of America; Tokyo Metropolitan Institute of Medical Science, Japan

## Abstract

Spontaneous fluctuations in blood oxygenation level-dependent (BOLD) images are the basis of resting-state fMRI and frequently used for functional connectivity studies. However, there may be intrinsic information in the amplitudes of these fluctuations. We investigated the possibility of using the amplitude of spontaneous BOLD signal fluctuations as a biomarker for cerebral vasomotor reactivity.

We compared the coefficient of variation (CV) of the time series (defined as the temporal standard deviation of the time series divided by the mean signal intensity) in two populations: 1) Ten young healthy adults and 2) Ten hypertensive elderly subjects with chronic kidney disease (CKD).

We found a statistically significant increase (P<0.01) in the CV values for the CKD patients compared with the young healthy adults in both gray matter (GM) and white matter (WM). The difference was independent of the exact segmentation method, became more significant after correcting for physiological signals using RETROICOR, and mainly arose from very low frequency components of the BOLD signal fluctuation (f<0.025 Hz). Furthermore, there was a strong relationship between WM and GM signal fluctuation CV's (R^2^ = 0.87) in individuals, with a ratio of about 1∶3.

These results suggest that amplitude of the spontaneous BOLD signal fluctuations may be used to assess the cerebrovascular reactivity mechanisms and provide valuable information about variations with age and different disease states.

## Introduction

Blood oxygenation level dependent (BOLD) is a complex signal arising from a combination of changes in cerebral blood volume (CBV), cerebral blood flow (CBF), and oxygen extraction fraction (OEF), leading to a change in the concentration of deoxyhemoglobin. Low frequency (f<0.1 Hz) spontaneous fluctuations in the BOLD signal have been observed in resting-state time series measurements [1]. These fluctuations appear to be correlated in functionally connected brain regions and form the basis of resting-state fMRI studies [2]–[4]. It has been shown that these fluctuations correlate with infraslow local field potential fluctuations at the recording sites with a delay comparable to the hemodynamic response [5]. Although the precise physiological origin of these signal fluctuations is not yet clear, they likely arise from oscillations in metabolic-linked brain physiology, arterial vasomotion, and hemodynamics [1], [6], [7], originating from myogenic and neurogenic sources [8]. They may also be sensitive to disease states. For example, previous research suggests that the amplitude of BOLD signal fluctuation changes in ischemic lesions [6], [9], [10]. In another study, Makedonov et al. recently showed that the BOLD signal in white matter can be used as a biomarker for aging and small vessel disease [11].

We view the spontaneous BOLD signal fluctuations as the response of the brain to the internal challenges to the cerebrovascular system, including heartbeat, inhalation, and baseline neuronal activity. We hypothesize that the response of the brain to these tiny challenges, as expressed by the normalized amplitude of the BOLD fluctuations, may provide information about cerebral perfusion, blood volume, oxygenation, and cerebrovascular autoregulatory mechanisms, in addition to neuronal activity. Consequently, these signals may yield insight into factors modulating the cerebrovasculature, such as aging and disease. To test this hypothesis, we compared the magnitude of spontaneous BOLD fluctuations in two groups who might be expected to have large differences in vasomotor reactivity [12]–[15]: young healthy adults and hypertensive elderly subjects with chronic kidney disease (CKD).

## Methods

### Ethics Statement

This prospective study was approved by the Stanford University's internal review board and was Health Insurance Portability and Accountability Act (HIPAA) compliant. Written informed consent was obtained prior to all human studies. The Stanford University's internal review board approved the consent procedure.

### Patient Population

Ten subjects (8 men and 2 women, age mean±std = 72±7 years; age [min max] = [56 83]) with hypertension and chronic kidney disease (CKD), defined as baseline estimated glomerular filtration rate (eGFR) <65 ml/min/1.73 m^2^ were recruited. Patients with diabetes or prior history of stroke were excluded from the study. Ten healthy sex-matched young volunteers (8 men and 2 women, age mean±std = 28±4 years; age [min max] = [24 35]) with no history of renal disease or hypertension were recruited in the study as a control group. Demographic information can be found in Table 1.

**Table 1:** Demographic information of the CKD and Control cohorts along with quantitative CV values calculated using different segmentation analysis approaches.

	Control	CKD
**Gender**	8 M/2 F	8 M/2 F
**Age, years (Range)**	28±4 (24–35)	72±7 (56–83)
**eGFR (ml/min)**	-	49.4±11.7
**Blood pressure (systolic), mmHg**	-	142±15
**Blood pressure (diastolic), mmHg**	-	74±10
**CV×10^−3^ (Method a, PVE), GM**	4.7±0.6	7.7±1.7
**CV×10^−3^ (Method a, PVE), WM**	3.2±0.4	4.5±0.8
**CV×10^−3^ (Method b, Strict Threshold), GM**	4.6±0.6	7.9±1.8
**CV×10^−3^ (Method b, Strict Threshold), WM**	3.0±0.4	4.2±0.7
**CV ×10^−3^ (Method c, Standard Template), GM**	4.3±0.5	5.5±1.3
**CV×10^−3^ (Method c, Standard Template), WM**	2.9±0.3	3.6±0.8
**CV×10^−3^ (Method b with RETROICOR), GM**	4.4±0.7	7.8±1.8
**CV×10^−3^ (Method b with RETROICOR), WM**	2.8±0.4	4.1±0.7

Methods a, b, and c refer to partial volume estimation (PVE), strict threshold and standard template methods, respectively. All values reported as mean±SD unless otherwise noted. Estimated glomerular filtration rate (eGFR) and systolic/diastolic blood pressures were measured within 90 days from the date of imaging.

### MR Protocol

Subjects were scanned at 3T (MR750, GE Healthcare, Waukesha, WI) using an 8-channel head coil. Resting-state BOLD signals were measured using a 2D gradient echo planar imaging (GRE-EPI) sequence (FOV = 22 cm, matrix = 64×64, slice thickness/slice spacing = 3.5/0 mm, number of slices = 35 covering the whole brain, TR/TE = 2 s/25 ms, flip angle = 75°, Number of time points = 180, imaging time: 6 min). A 3D T1-weighted image was also acquired for anatomic reference using an IR-SPGR sequence covering the entire brain (TR/TE/TI = 8.18/3.2/900 ms, matrix = 256×256, in-plane resolution = 0.94×0.94 mm, slice thickness = 1 mm, 176 sagittal slices). Cardiac and respiratory functions were monitored using the scanner's built-in photoplethysmograph and respiratory belt.

### Post-processing

After reconstruction, EPI images were corrected for movement using least-squares minimization. The 3D T1-weighted image was co-registered to the EPI images and normalized to the Montreal Neurological Institute (MNI) template. Using the obtained transfer matrix, EPI images were also registered to the standard MNI space. These processing steps were carried out using the FSL software package (http://www.fmrib.ox.ac.uk/fsl). To further reduce any movement-related signal fluctuations, 6 rigid-body movement parameter time-courses (estimated in the motion correction step) were removed from the data by linear regression. Baseline scanner drifts were estimated and removed from the EPI images by first-order polynomial detrending. The coefficient of variation (CV) of signal fluctuation, defined as the temporal standard deviation of the EPI signal amplitudes normalized by the mean signal intensity, for each voxel was then calculated. To calculate the mean CV in GM and WM, we performed 3 different analyses to evaluate the sensitivity of the results to the segmentation process:

a) Partial Volume Estimation (PVE) Method: GM and WM partial volume maps were estimated from the T1-weighted structural images in each voxel. Each voxel was assigned a value between 0 and 1 that represents the proportion of GM or WM present in that given voxel [16] using the automated segmentation tool of FSL (FAST). Mean CV in GM and WM were calculated using Eq. 1: 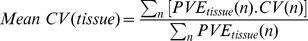
(Eq. 1)where PVE is the partial volume estimated for a particular tissue type and *n* is the number of voxels in the brain.

b) Strict Threshold Method: GM and WM masks were generated from the GM and WM PVE maps including only voxels with a partial volume threshold of >0.8. Only voxels that had PVE levels above this threshold were included in the analysis.

c) Standard Template Method: After placing the images into MNI atlas space using FSL, ICBM152 nonlinear GM and WM standard templates (McConnell Brain Imaging Centre, Montreal Neurological Institute, McGill University) were used for the GM and WM regions-of-interest.

Given that resting-state fMRI data is often post-processed using low-pass filtering to minimize the effects of cardiac and respiratory variation, we examined the effect of different types of filtering. First, we studied the signal without any filtering, assuming there may be important and potentially useful information in the higher frequency bands. We also examined the results using three different band-pass filters (0.01–0.014 Hz, 0.014–0.025 Hz and 0.025–0.1 Hz) to estimate the contribution of these frequency bands to the BOLD signal fluctuations. These bands were chosen based on manual inspection of the frequency spectrum of the BOLD time series to capture the fast initial decay of the signal in the frequency domain.

Pulsatility of blood flow in the brain and respiration-induced magnetic field changes or motion can cause appreciable modulation of the BOLD signal [17]. To evaluate the contribution of these effects on the CV of signal fluctuations, we performed a separate analysis after removing the physiological motion effects from reconstructed images utilizing the RETROICOR method [17], based on the recorded cardiac and respiratory time-courses. RETROICOR fits a low-order Fourier model to the BOLD time series based on the time of each image acquisition relative to the phase of the cardiac and respiratory cycles for each slice. These effects are subsequently removed from of the BOLD time series using regression. Since RETROICOR is based on the assumption of quasi-periodic variation of physiological noise, cardiac and respiratory time-courses were also inspected for any sign of arrhythmia.

Although the effect of motion upon the CV is corrected for by the initial step of motion correction and also by regressing the motion parameters, we also compared the estimated average displacement from the 6 rigid-body movement parameters for each subject before correction.

### Statistical Analysis

To evaluate the statistical significance of the analyses we performed a Wilcoxon test, a nonparametric test for equality of population medians of two independent cohorts. The null hypothesis was that CV values in control and CKD cohorts are independent random samples with equal median. The alternative hypothesis was that the medians of these two random variables were not equal. A threshold of p<0.05 was considered significant.

## Results

**Figure 1:**
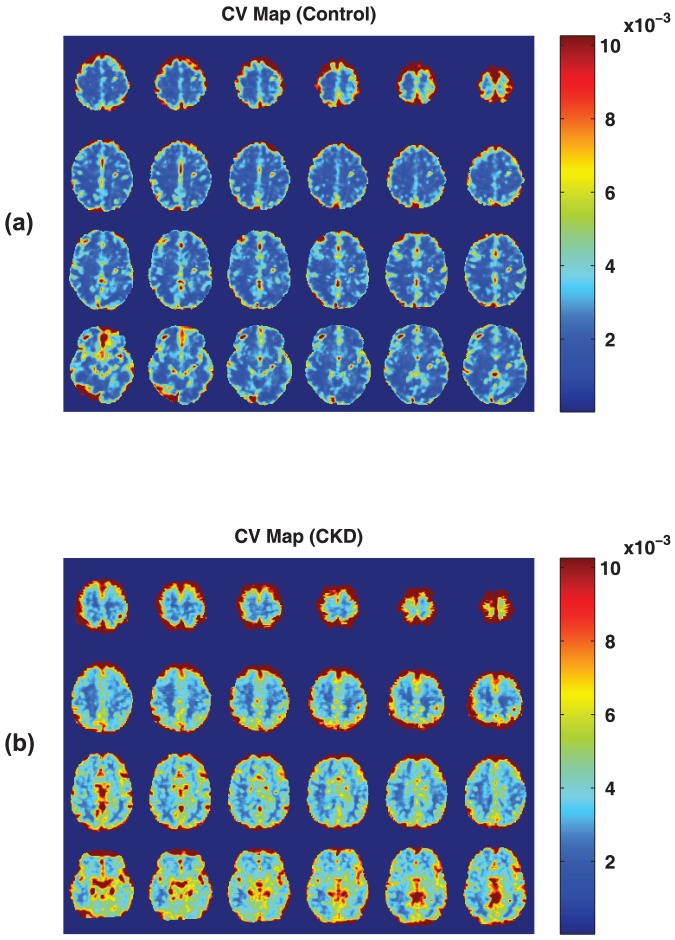
Coefficient of variation (CV) maps calculated for a representative (a) CKD patient and (b) young healthy control. No temporal filtration was applied.

**Figure 2:**
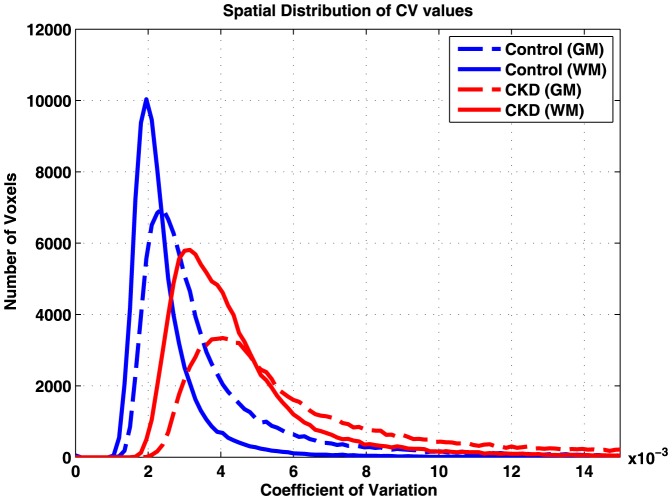
CV histograms in GM (dotted lines) and WM (solid lines) for a representative CKD patient (red) and young healthy control (blue), corresponding to the CV maps shown in Figure 1. GM and WM ROIs were defined using the strict threshold approach.

**Figure 3:**
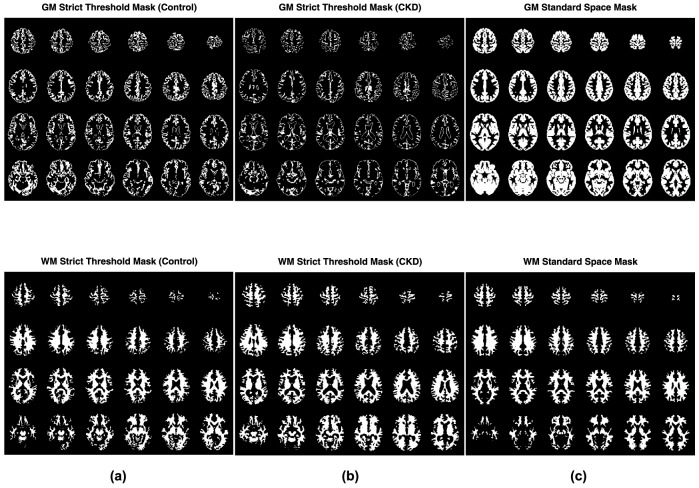
GM (top rows) and WM (bottom rows) masks used for calculating mean CV in these two tissue types: The GM and WM threshold masks derived from partial volume estimation (PVE) maps (PVE threshold>0.8) for representative (a) control and (b) CKD subjects. ICBM152 standard GM and WM template masks are shown in column (c), and were, of course, independent of the cohort group.

Figures 1 and 2 show CV maps and the histogram of the CV values obtained in representative control and CKD subjects. Contrast between GM and WM can be seen in both cohorts; however, the average CV in both GM and WM is higher in CKD subjects compared to that of the control population. For each subject, mean CV values in GM and WM were calculated using the 3 different methods for GM/WM segmentation. Figure 3 shows the GM and WM threshold masks for the subjects shown in Figure 1 using the different approaches described earlier.

**Figure 4:**
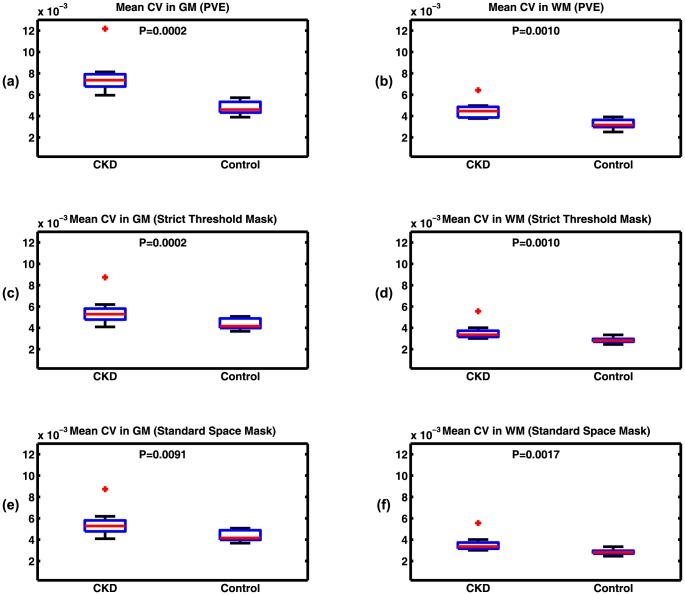
Mean CV of BOLD signal fluctuations in GM (left columns) and WM (right columns) for young healthy control and elderly CKD populations using different approaches: (a–b) PVE, (c–d) strict threshold masks, and (e–f) ICBM152 standard template masks. Mean CV is significantly higher in the CKD group in both tissue types using all approaches. The difference is more significant using PVE and the strict threshold masking approaches compared to the standard template approach.

Mean CV in GM and WM masks, calculated using these 3 methods for all subjects, is presented as Figure 4. There is significantly higher mean CV of fluctuations in the elderly CKD cohort in both GM and WM for all segmentation approaches. The results of PVE and strict threshold masking approaches (methods a and b) are similar and are more significantly different compared to the standard template approach (method c). For all the subsequent analyses we quote values based on the strict threshold segmentation approach (method b). Table 1 summarizes the quantitative CV values in the two cohorts using the different segmentation methods.

**Figure 5:**
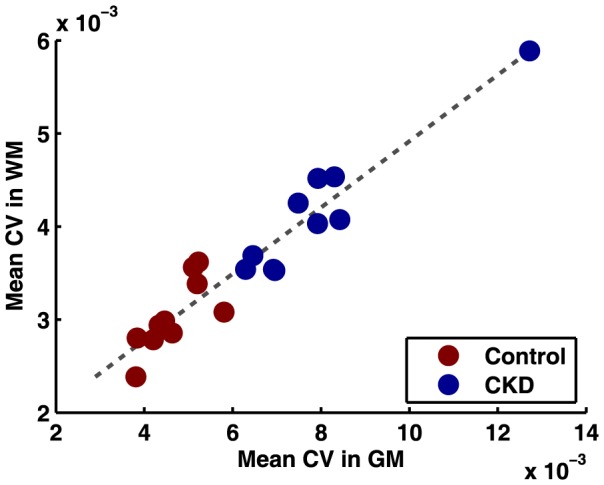
Scatterplot of mean CV in GM and WM for CKD (red) and Control (blue) populations. The dotted line represents a linear fit of the data (y = 0.36×+0.0014, R^2^ = 0.87).

The relationship between individual CV values in GM and WM for all subjects is presented in Figure 5. There is a strong linear relationship between CV values in GM and WM in both populations, with the elderly CKD patients lying largely above the young normal subjects. The slope of a linear fit to the data was 0.36 (y = 0.36×+0.0014, R^2^ = 0.87).

**Figure 6:**
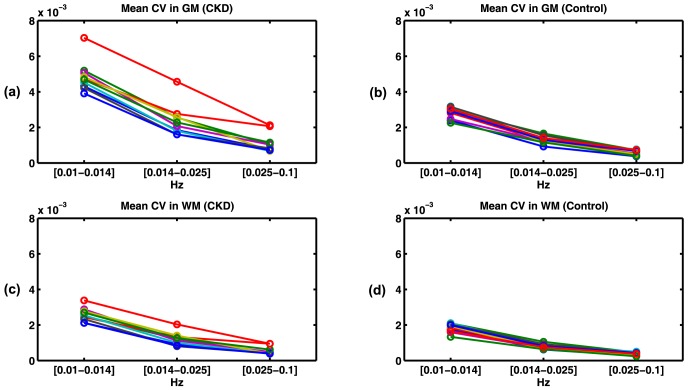
Mean CV of signal fluctuation in GM (a,b) and WM (c,d) in different frequency intervals ([0.01–0.014] Hz, [0.014–0.025] Hz and [0.025–0.1] Hz) for CKD (a,c) and control (b,d) cohorts. Lower frequency components have a larger contribution to the BOLD signal fluctuations.

The results of the effects of the three different band-pass filters (0.01–0.014 Hz, 0.014–0.025 Hz and 0.025–0.1 Hz) on the mean CV in GM and WM are shown in Figure 6. It can be seen that the changes in the BOLD signal fluctuations mainly arise from very low frequency components (<0.025 Hz). Lower frequency components showed higher contribution to the overall signal fluctuation.

**Figure 7:**
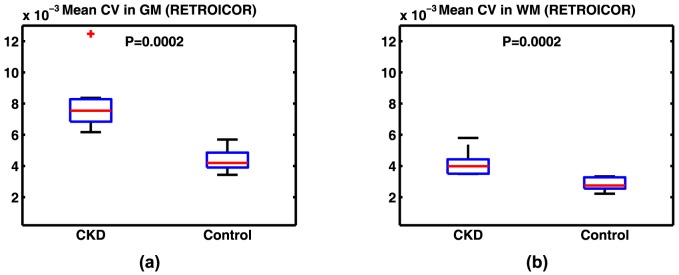
Mean CV of signal fluctuations in (a) GM and (b) WM for young healthy control and CKD populations after applying RETROICOR. Results are not significantly different from those achieved without RETROICOR (Figure 3c–d).

**Figure 8:**
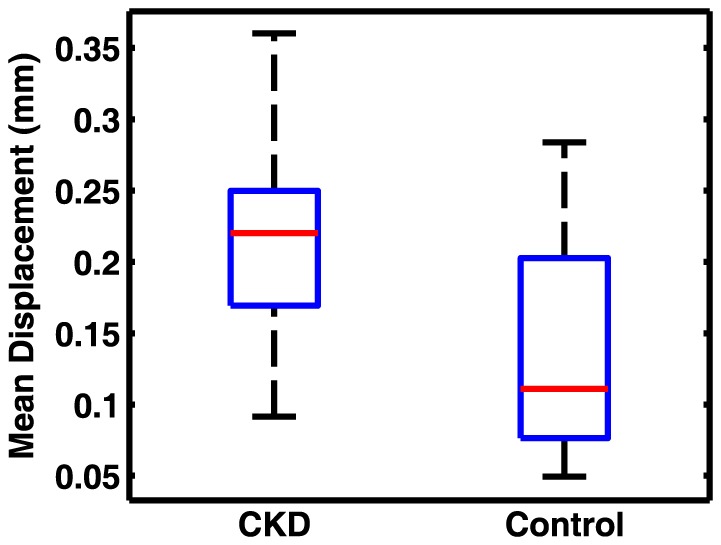
Mean head displacement (mm) before correction during the scan for CKD and control cohorts (p = 0.07).

**Figure 9:**
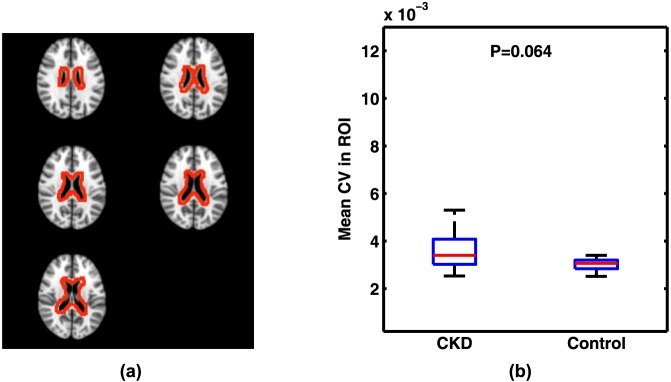
Mean CV measured in an area possibly susceptible to non-rigid body movements in the brain (interface between the lateral ventricles and brain parenchyma). (a): The ROI used for calculations. (b): Mean CV values calculated in the ROI.

In a separate analysis of the data, we calculated the CV's in GM and WM after applying the RETROICOR technique to remove the effects of cardiac and respiratory motion (Figure 7). After applying RETROICOR, the difference in CVs in GM and WM became slightly larger between the two cohorts, though the increase itself due to RETROICOR was not significant. The quantitative CV values for this analysis are also summarized in Table 1. Differences in mean head displacement before correction in the two cohorts is shown as Figure 8. Although the movement in the elderly CKD patients before correction is higher (mean difference = 0.07 mm), it is not statistically different from the normal young subjects (P = 0.07). We expect this difference to be even less significant after the two-step motion correction used in this study. To evaluate the sensitivity of our measurements to non-rigid movements in the brain caused by heartbeat and respiration, we also calculated mean CV in the WM area in voxels immediately adjacent to the lateral ventricles. For this analysis, the region of interest (ROI) was manually drawn on the T1 weighted images in the MNI space (Figure 9a). Mean CV values calculated within the ROI in control and CKD cohorts are presented in Figure 9b. Average CV values obtained within the ROI in control and CKD cohorts were 3.0×10^−3^ and 3.5×10^−3^, respectively. These values were similar to those measured in WM using the standard template method (2.9×10^−3^ and 3.6×10^−3^). The difference between the two cohorts, however, was less significant within this ROI (P = 0.064) compared to that of the whole WM (P = 0.0017). This indicates that the reported difference of the CVs between the two cohorts does not arise from non-rigid body movements in the brain.

## Discussion

In this study we compared the mean CV values in GM and WM between young normal and elderly, hypertensive CKD patients. Our results indicate a statistically significant increase in BOLD signal fluctuations in the CKD cohort and also between GM and WM within each cohort. We also investigated the origin of the fluctuations employing different analysis schemes. Using different GM/WM masking approaches, we evaluated the sensitivity of the results to the particular segmentation method. Using the strict threshold mask, there were different numbers of GM and WM voxels for different subjects; however, since the number of voxels was large (>2000) for all subjects, we believe that the difference in the number of voxels did not affect our estimation of the mean CVs. The fact that the strict threshold and PVE masking approaches generated very similar results also supports this assumption. The linear registration algorithm for transferring the data from the imaging space to the standard atlas was imperfect, particularly for the CKD patients, due to their larger CSF compartment. Therefore, the results of using the standard templates were slightly different from the PVE and strict threshold masking approaches. Another factor that can potentially affect the accuracy of the WM/GM segmentation in this study is the presence of white matter hyperintensities (WMHs), which are often associated with aging and can be segmented as GM using the segmentation tools [11]. In this study, the segmentation results were inspected manually and we observed no significant segmentation error. Due to large number of voxels in GM and WM, however, we do not expect minor segmentation errors to significantly affect the calculation of mean CVs. Particularly; the segmentation method C (“Standard Template Method”) that used a standard template would not erroneously misclassify WMHs as gray matter. The fact that all methods used for calculating mean CVs in GM/WM demonstrated a significant difference between the two cohorts indicates that these results are somewhat independent of the precise segmentation strategy employed.

It is interesting that the WM to GM signal fluctuation lie on a line (Figure 5). The slope is close to the ratio of GM and WM cerebral blood volume (CBV) reported in the literature [18], [19]. This may suggest that CBV is a major contributor to the fluctuations of the BOLD signal. However, it is interesting to note that most prior studies suggest that CBV decreases with age [19], suggesting that other hemodynamic factors such as changes in OEF or neuronal activity may also play a role in these findings. OEF is thought to increase with age [19], thus potentially offsetting the effects of CBV changes, if any. Grady et al. [20] demonstrated that there is an age-related decline in the ability to suspend non-task-related (i.e., resting state) brain activity and engage areas for carrying out memory tasks. This could be due to increased resting-state brain activation in the older population and can also be a contributing factor in higher BOLD signal fluctuations seen in the elderly CKD population. Since neuronal activity mainly arises from the GM, the presence of a significant difference of the CV values in WM as well as GM, suggests that the difference in the CV of the spontaneous BOLD signal fluctuation mainly originates from a difference in the vascular reactivity rather than neuronal activity. Differences in the baseline neuronal activity, however, might be the cause of a more significant difference in the CV values in GM compared to that of WM.

To investigate the possibility of the difference of the CVs originating from physiological motion or respiration-induced magnetic field changes, we used RETROICOR to reduce the effects of these confounding components [17]. Using RETROICOR resulted in more pronounced signal differences between the two cohorts. This change, however, was not large, suggesting that recording the respiratory and cardiac physiological signals may not be necessary in other similar applications and analyses. Although rigid body movements were used as a regressor in our analyses, motion correction algorithms cannot completely correct for non-rigid body movements of the brain due to heartbeat and respiration. But since these movements mainly appear at the CSF boundaries on the surface of the brain (note the high CV values on the surface in Figure 1), they are largely eliminated from the analysis for calculating CVs in GM and WM. In addition these voxels represent a very small fraction of voxels included in the analysis.

Our analysis indicated that most of the BOLD signal fluctuations originated from very low frequency components (f<0.025 Hz). Using a reflected light imaging technique, it has been suggested that cerebral vasomotion demonstrates a 0.1 Hz oscillation [8]. The discrepancies can be explained by the difference in the point spread function of BOLD imaging as compared to that of the reflected light imaging as well as contributions from sources other than vasomotion, such as neuronal modulation. The long TR (2 s) used in this study does not allow for a true frequency analysis of the BOLD signal fluctuation power spectrum, due to the aliasing of the cardiac variations. Since the TR was considerably longer than the cardiac period (∼0.9 s), one possibility is that the low-frequency fluctuations may in fact reflect the aliased response to cardiac fluctuations. Although, considering the frequency of the cardiac waveforms in our experiment (1.13+/−0.13 Hz) and the sampling frequency (0.5 Hz) lower frequency components (f<0.025) probably do not result from an aliasing of the cardiac peak frequency or its first 2 harmonics. With a shorter TR (∼400 ms), it would be possible to differentiate and compare the spontaneous neural activity, respiration, and cardiac response components between two populations, which are largely aliased into the low frequency band using longer TRs. Shorter TR scans would result in lower SNR images and limited number of slices resulting in partial brain coverage. Approaches to counter these problems include multiband imaging [21] and the use of higher magnetic fields, such as 7 T [22]. Higher BOLD signal fluctuation in the elderly CKD population could be due to higher baseline concentrations of deoxyhemoglobin, difference in vascular compliance, increased instability of autoregulatory mechanisms, increased neuronal metabolic activity, and/or CSF contamination due to atrophy in these patients. Baseline oxygenation differences could be assessed using MR-based oxygenation methods [23] or PET [19]. Given that the differences between the cohorts are seen in both WM and GM, it is unlikely that differences in CSF partial volume are solely responsible.

A major limitation of this study is that we are not able to distinguish the possibly separate effects of aging, hypertension, and CKD on the signal fluctuations. The two cohorts were chosen based on the expectation that there would be a potentially large difference in arterial vasomotor function, based on prior work [12]–[15], [24]. Further studies on healthy elderly populations, elderly hypertensive populations without CKD, and younger hypertensive patients will be required to assess the relative contributions of these various factors. However, these initial results suggest that spontaneous BOLD signal fluctuation amplitude may represent a unique source of contrast that is sensitive to cerebrovascular compensatory mechanisms.
